# The hepatic microenvironment promotes lung adenocarcinoma cell proliferation, metastasis, and epithelial–mesenchymal transition via METTL3-mediated N6-methyladenosine modification of *YAP1*

**DOI:** 10.18632/aging.202397

**Published:** 2021-01-20

**Authors:** Xue-Feng Ni, Quan-Qin Xie, Jie-Min Zhao, Yan-jie Xu, Mei Ji, Wen-Wei Hu, Jun Wu, Chang-Ping Wu

**Affiliations:** 1 Department of Oncology, The Third Affiliated Hospital of Soochow University, Changzhou, China; 2 Department of Gastroenterology, The Third Affiliated Hospital of Soochow University, Changzhou, China

**Keywords:** adenocarcinoma, liver inflammatory microenvironment, METTL3, m6A, YAP1

## Abstract

The inflammatory microenvironment plays an important role in the onset and progression of lung adenocarcinoma (LUAD), and the liver is a suitable site of metastasis for LUAD cells. However, whether the inflammatory microenvironment of the liver is conducive to the proliferation, invasion, and metastasis of LUAD cells remains unclear. In this study, we confirmed that the hepatic inflammatory microenvironment stimulated by IL-6 promoted the proliferation, migration, invasion, and epithelial–mesenchymal transition of LUAD cells, increased the m6A methylation of total RNA, and transcriptionally activated *METTL3* expression. Additionally, METTL3 activated the YAP1/TEAD signaling pathway by increasing the m6A modification and expression of *YAP1* mRNA. These results indicate that the hepatic inflammatory microenvironment plays a role in regulating the biological functions of LUAD cells. Further, our study identifies a molecular mechanism that may provide a new strategy for the early diagnosis, treatment, and prognosis of liver metastasis in LUAD patients.

## INTRODUCTION

Lung cancer is one of the most common malignant tumors worldwide and is the most frequently diagnosed fatal tumor type in China [1, 2]. Although great progress has been made in the treatment strategies for lung cancer, such as surgical resection, radiotherapy, and chemotherapy, 5-year survival remains low at approximately 17% [3]. The liver is one of the most common sites of lung cancer metastasis. The incidence of liver metastasis in lung cancer patients is 38%–44%, and the incidence of liver metastasis at lung cancer autopsy is 40%–61% [4]. Liver metastasis of lung cancer has a poor prognosis, and most patients die within 7 months mainly because of liver failure, rupture or hemorrhage of metastatic liver tumors, and portal vein tumor thrombus [5]. Therefore, elucidating the biological mechanism of metastasis is essential to identify new and more effective treatments.

Increasing evidence indicates that the inflammatory microenvironment plays an important role in the onset and progression of cancer and that the signal pathways mediated by inflammatory cytokines participate in the malignant transformation of tumor cells [6]. The inflammatory tumor microenvironment is mainly composed of tumor cells, interstitial fibroblasts, immune cells, various inflammatory factors, chemokines, and growth factors [7, 8]. In the inflammatory microenvironment of tumors, several cell signaling pathways induce the expression of proinflammatory factors, such as interleukin (IL)-1, IL-6, tumor necrosis factor alpha (TNFα), transforming growth factor beta (TGFβ), chemokines, and vascular growth factors, promote the proliferation, invasion, and metastasis of tumor cells, and increase angiogenesis [9].

Human IL-6 is a proinflammatory cytokine produced by diverse cell types [10]. Anti-IL-6 therapy neutralizes IL-6 and inhibits the proliferation of tumor cells in early-stage diseases. As a result, the use of IL-6 inhibitors in the prevention and treatment of tumors has become a research topic of high interest [11]. Studies have shown that there is active expression and increased secretion of IL-6 in or around tumor tissues. By changing the microenvironment of tumor tissues, IL-6 promotes the abnormal proliferation of normal tissue cells and expression of peripheral vascular genes, leading to the development and metastasis of malignant tumors [12]. Several IL-6 inhibitors, including the anti-IL-6 monoclonal antibody siltuximab and the anti-IL-6 receptor monoclonal antibody tocilizumab, have been developed [13, 14]. Our previous study found that CRP/Alb was an independent risk factor for survival in patients with advanced lung cancer and that the CRP/Alb prognostic score ratio was a better predictor than CRP, GPS, mGPS, NLR, PLR, and MLR [15]. In addition, IL-6 (10 ng/ml) significantly stimulated the secretion of CRP protein in a co-culture system of hepatocytes and lung adenocarcinoma (LUAD) cells and significantly promoted the proliferation and invasion of A549 and H1975 LUAD cells. Therefore, inflammatory microenvironments with high IL-6 expression may play an important role in regulating the liver metastasis of lung cancer cells.

N6-methyladenosine (m6A) methylation is an important RNA modification that primarily occurs at the sixth site of adenine (A) in mRNA and long non-coding RNA (lncRNA) [16, 17]. Several studies showed that different levels of m6A methylation are related to the self-renewal of tumor stem cells and the growth, proliferation, chemoresistance, and radiosensitivity of cancer cells [18–23].

The inflammatory microenvironment plays an important role in the development of LUAD, and the liver is a suitable site of metastasis for LUAD cells. However, whether the inflammatory microenvironment of the liver contributes to the proliferation, invasion, and metastasis of LUAD cells remains unclear. To address this, the current study examined the role of the hepatic inflammatory microenvironment on lung cancer cells, focusing on the effect of m6A methylation on the biological functions of LUAD cells. By studying the related molecular mechanisms, we aimed to provide new research strategies for the early diagnosis, treatment, and prognosis of liver cancer metastasis in lung cancer patients.

## RESULTS

### The hepatic inflammatory microenvironment promotes the proliferation, migration, and invasion of LUAD cells

**Figure 1:**
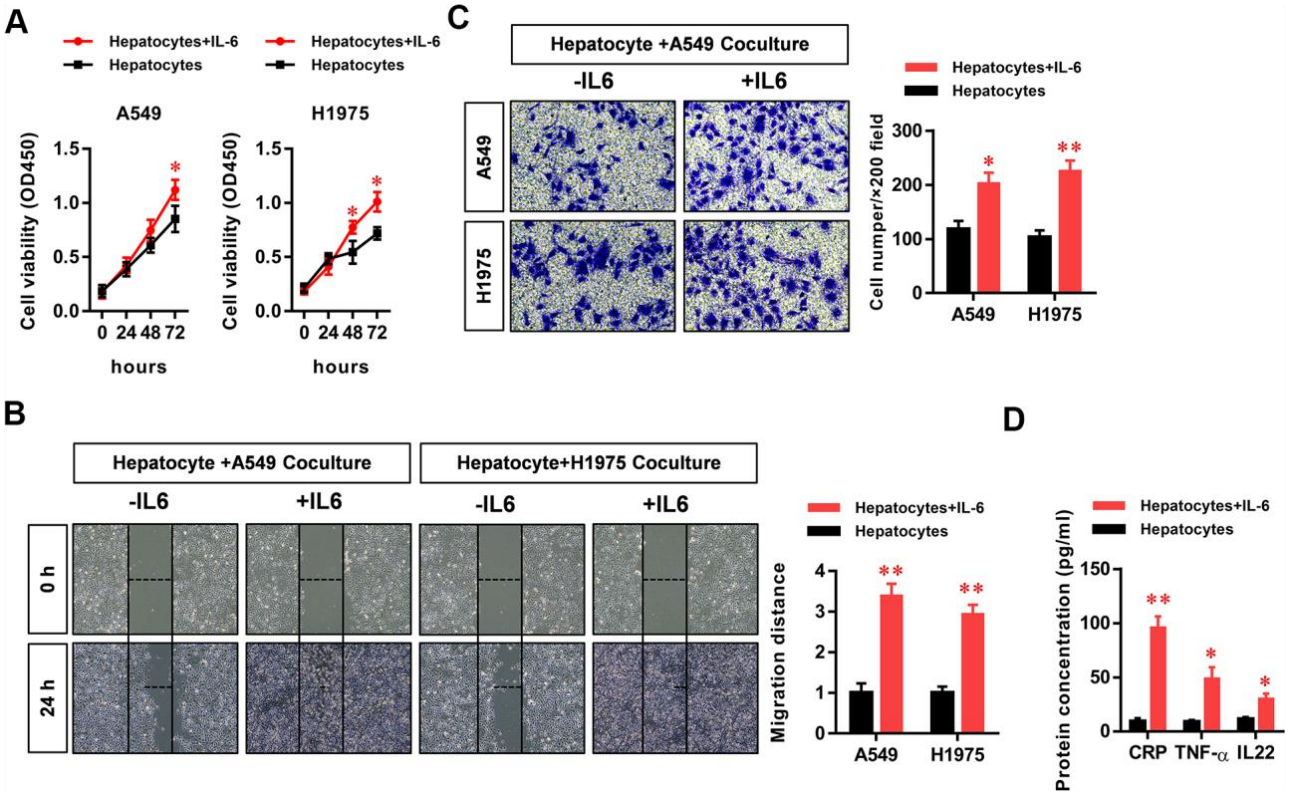
**The hepatic inflammatory microenvironment promoted the proliferation, migration, and invasion of lung adenocarcinoma (LUAD) cells.** The IL-6-stimulated inflammatory microenvironment simulated by liver cells (our hepatic inflammatory microenvironment model) significantly promoted the (**A**) proliferation, (**B**) migration, and (**C**) invasiveness of A549 and H1975 LUAD cells and (**D**) significantly increased the secretion of the primary hepatocyte inflammatory factors CRP, TNFα, and IL-22 (*P<0.05, **P<0.01 versus the control group).

To study the effect of the hepatic inflammatory microenvironment on the biological function of LUAD cells, we added IL-6 to a co-culture system of hepatocytes and LUAD cells to simulate the hepatic immune microenvironment. Using CCK8 assays, we found that IL-6 stimulated the inflammatory microenvironment simulated by hepatocytes (our model of the inflammatory microenvironment in the liver) to significantly promote the proliferation of LUAD cells (Figure 1A). In addition, we found that the hepatic inflammatory microenvironment significantly promoted the migration (Figure 1B) and invasion (Figure 1C) abilities of LUAD cells. In addition, the results of ELISA experiments revealed the significantly increased expression of CRP, TNF-α, and IL-22 cytokines in the inflammatory microenvironment of the liver (Figure 1D).

### The hepatic inflammatory microenvironment promotes the epithelial–mesenchymal transition (EMT) of LUAD cells

**Figure 2:**
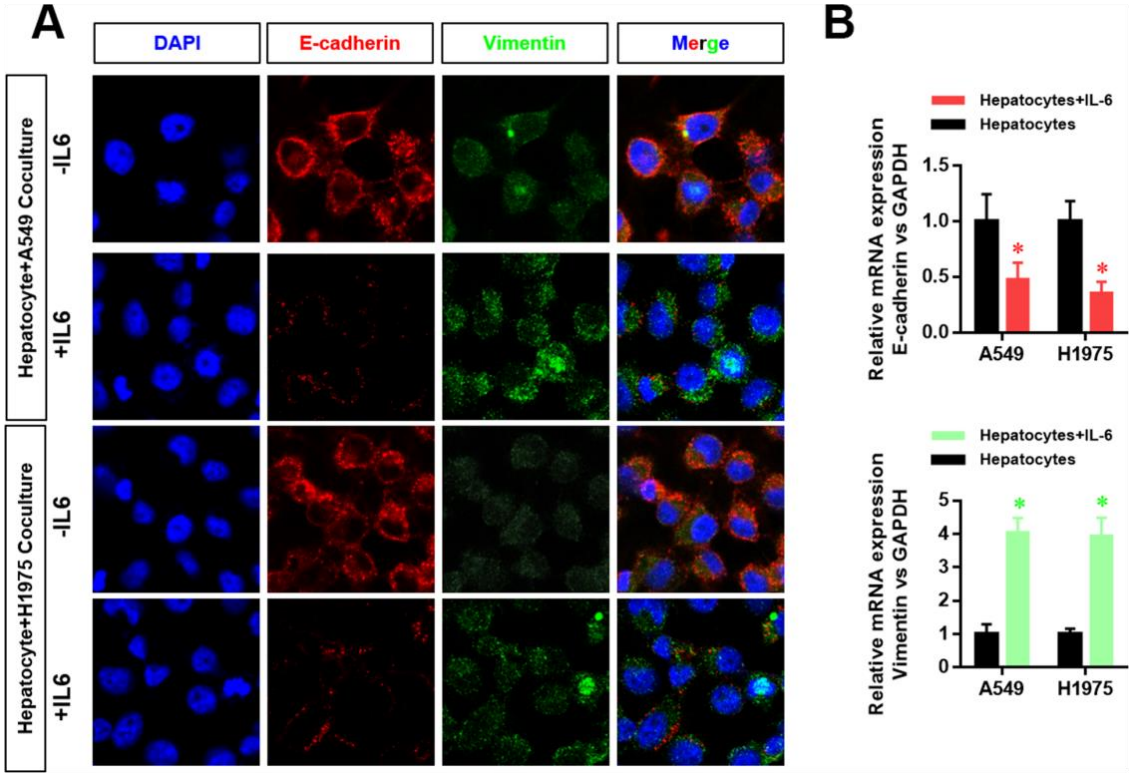
**The hepatic inflammatory microenvironment promoted the epithelial–mesenchymal transition (EMT) of lung adenocarcinoma cells.** (**A**) The expression level of E-cadherin and vimentin in A549 and H1975 cells was detected by laser confocal scanning microscopy. (**B**) Changes in *CDH1* and *VIM* mRNA expression in A549 and H1975 cells were detected by real-time PCR (*P<0.05 versus the control group).

The EMT phenotype is an important indicator of tumor cell metastasis. Therefore, we further examined the effect of the inflammatory microenvironment in the liver on the EMT of LUAD cells. We found that the hepatic inflammatory microenvironment significantly inhibited the expression of E-cadherin and increased the expression of vimentin in LUAD cells. Furthermore, the expression of E-cadherin (*CDH1*) and vimentin (*VIM*) in LUAD cells was verified by RT-PCR (Figure 2).

### The hepatic inflammatory microenvironment significantly increased m6A methylation and METTL3 expression in A549 and H1975 LUAD cells

**Figure 3:**
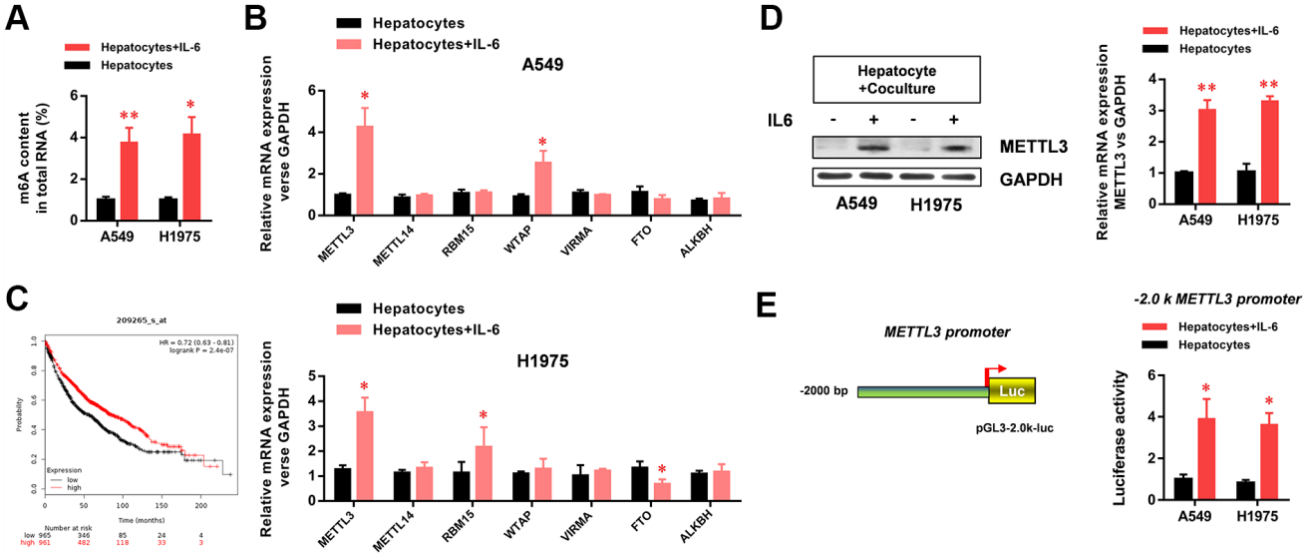
**The hepatic inflammatory microenvironment significantly increased m6A methylation and METTL3 expression in A549 and H1975 lung adenocarcinoma (LUAD) cells.** (**A**) The hepatic inflammatory microenvironment significantly increased the level of m6A methylation in A549 and H1975 LUAD cells. (**B**) *METTL3* mRNA expression in A549 and H1975 cells was significantly increased by the inflammatory microenvironment of the liver. (**C**) The Kaplan–Meir Plot database indicated that *METTL3* expression in lung cancer was positively correlated with prognosis. (**D**) The hepatic inflammatory microenvironment significantly increased METTL3 expression in A549 and H1975 cells. (**E**) The hepatic inflammatory microenvironment significantly increased the activity of the *METTL3* promoter in A549 and H1975 cells (*P<0.05 versus the control group).

To further study the mechanism by which the hepatic inflammatory microenvironment regulates the biological function and EMT of LUAD cells, we detected the methylation levels of m6A (N6 methyladenine, 6-methyladenine) in LUAD cells. M6A is the most common methylation modification in the internal sequence of eukaryotic mRNA, and it functionally regulates the transcriptome of eukaryotic cells to modulate the splicing, enucleation, localization, translation, and stabilization of mRNA. The results showed that the m6A methylation levels of total RNA in LUAD cells were significantly increased in the hepatic inflammatory microenvironment (Figure 3A). In addition, we screened m6A-related methyltransferases (*METTL3*, *METTL14*, *RBM15*, *WTAP*, *VIRMA*, *FTO*, and *ALKBH*) by RT-PCR. The results showed that *METTL3* expression in LUAD cells was significantly increased in the inflammatory microenvironment of the liver (Figure 3B). Because METTL3 is the critical enzyme of m6A methylation, we next analyzed *METTL3* expression data in the Kaplan–Meier Plotter database. The results showed that high *METTL3* expression levels in lung cancer were significantly correlated with poor patient prognosis (Figure 3C). We further verified the increased protein expression of METTL3 in LUAD cells in the hepatic inflammatory microenvironment (Figure 3D). Using luciferase reporter assays, we demonstrated that the promoter activity of *METTL3* in LUAD cells was significantly increased in the hepatic inflammatory microenvironment (Figure 3E).

### METTL3 elevates YAP1 gene expression in A549 and H1975 LUAD cells via m6A methylation

**Figure 4:**
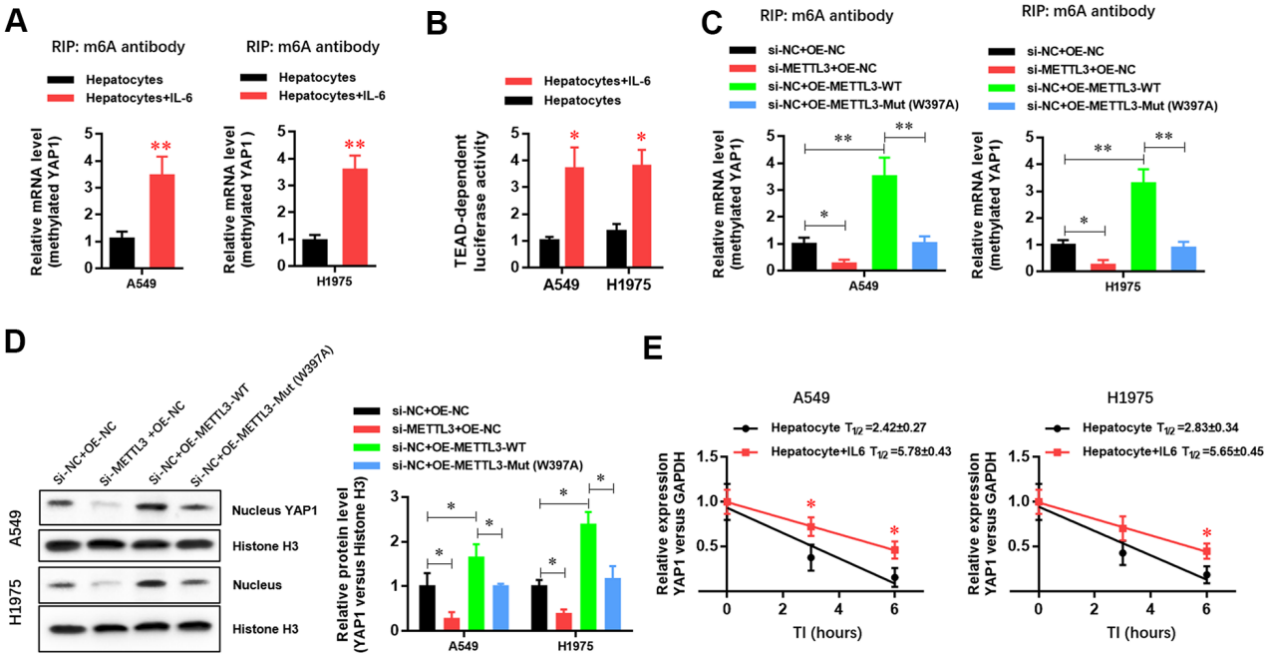
**METTL3 elevated *YAP1* expression in A549 and H1975 lung adenocarcinoma (LUAD) cells via m6A methylation.** (**A**) RIP experiment showing that the hepatic inflammatory microenvironment significantly increased the m6A methylation of *YAP1* in A549 and H1975 LUAD cells. (**B**) TEAD luciferase reporter assays showed that the inflammatory microenvironment of the liver significantly increased YAP1 signaling in A549 and H1975 cells. (**C**) RIP experiment showing that *METTL3* gene silencing significantly inhibited the m6A methylation of *YAP1*, and METTL3 overexpression significantly increased the m6A methylation of *YAP1*. However, mutating a critical active site in METTL3 (W397A) had no effect on the m6A methylation of *YAP1*. (**D**) Western blot experiment showing that *METTL3* gene silencing significantly inhibited the nuclear expression level of YAP1 protein, and the overexpression of METTL3 significantly increased the nuclear expression level of YAP1 protein. However, mutating the key active site of METTL3 (W397A) had no effect on YAP1 protein expression levels. (**E**) The hepatic inflammatory microenvironment significantly increased the stability of *YAP1* mRNA in A549 and H1975 cells (*P<0.05, **P<0.01 versus the control group).

To further explore the molecular mechanism by which the inflammatory microenvironment of the liver regulates the biological function of LUAD cells through METTL3, we conducted Me-RIP experiments and found that the m6A methylation level of *YAP1* mRNA in LUAD cells was significantly increased (Figure 4A). The luciferase assay results also showed that the activity of the YAP1/TEAD pathway was significantly increased (Figure 4B). To verify the role of METTL3 in regulating the m6A methylation and expression of YAP1 mRNA, we generated *METTL3*-specific siRNA, a wild-type METTL3 overexpression vector, and an enzyme activity-specific deletion mutant METTL3 overexpression vector. The results showed that *METTL3* downregulation significantly inhibited the expression of *YAP1* mRNA in LUAD cells (Figure 4C). Furthermore, the overexpression of wild-type METTL3 significantly increased *YAP1* mRNA expression in LUAD cells (Figure 4C). However, in the absence of methylase activity, METTL3 had no significant effect on *YAP1* mRNA expression in LUAD cells (Figure 4C), suggesting that the m6A methylase activity of METTL3 played a critical role in regulating the expression of *YAP1* mRNA. Western blotting experiments further verified the effect of METTL3 on YAP1 protein expression (Figure 4D). In an mRNA decay experiment with cells cultured in the presence of actinomycin D (a transcription inhibitor), we further demonstrated that the decay rate of *YAP1* mRNA in the hepatic inflammatory microenvironment was significantly reduced, whereas its stability was increased (Figure 4E). The results showed that the hepatic inflammatory microenvironment was modified by the METTL3-mediated m6A methylation of *YAP1* mRNA, which enhanced the stability of *YAP1* mRNA, increased YAP1 expression, and promoted the activity of the YAP1/TEAD signaling pathway.

### YAP1 inhibition significantly abolished the effects of the hepatic inflammatory microenvironment on the proliferation, migration, and invasion of LUAD cells

**Figure 5:**
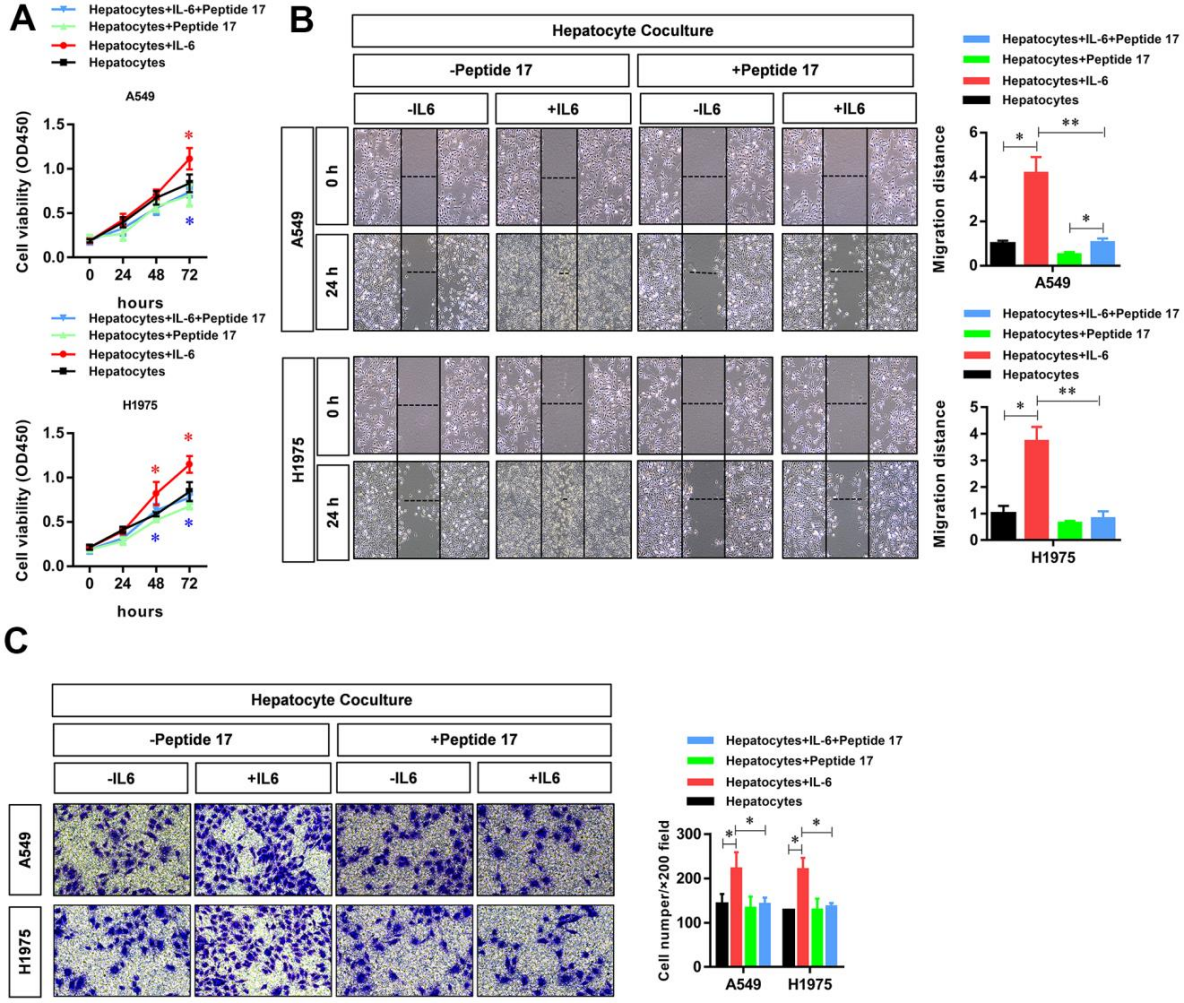
**YAP1 blockade significantly inhibited the ability of the hepatic inflammatory microenvironment to promote the proliferation, migration, and invasion of lung adenocarcinoma cells.** Peptide 17 significantly inhibited the ability of the inflammatory microenvironment of the liver to promote the (**A**) proliferation, (**B**) migration, and (**C**) invasiveness of lung adenocarcinoma cells (*P<0.05, **P<0.01 versus the control group).

To verify the critical role of the YAP1/TEAD signaling pathway in regulating the biological functions of LUAD cells in the hepatic inflammatory microenvironment, we treated LUAD cells with peptide 17, a specific inhibitor of the YAP1/TEAD signaling pathway. The results showed that the inflammatory microenvironment of the liver failed to significantly promote the proliferation (Figure 5A), migration (Figure 5B), and invasion (Figure 5C) of LUAD cells, indicating that the YAP1/TEAD signaling pathway plays an essential role in promoting tumorigenesis in the hepatic inflammatory microenvironment.

### YAP1 inhibition significantly abolished the activation of EMT in LUAD cells by the hepatic inflammatory microenvironment

**Figure 6:**
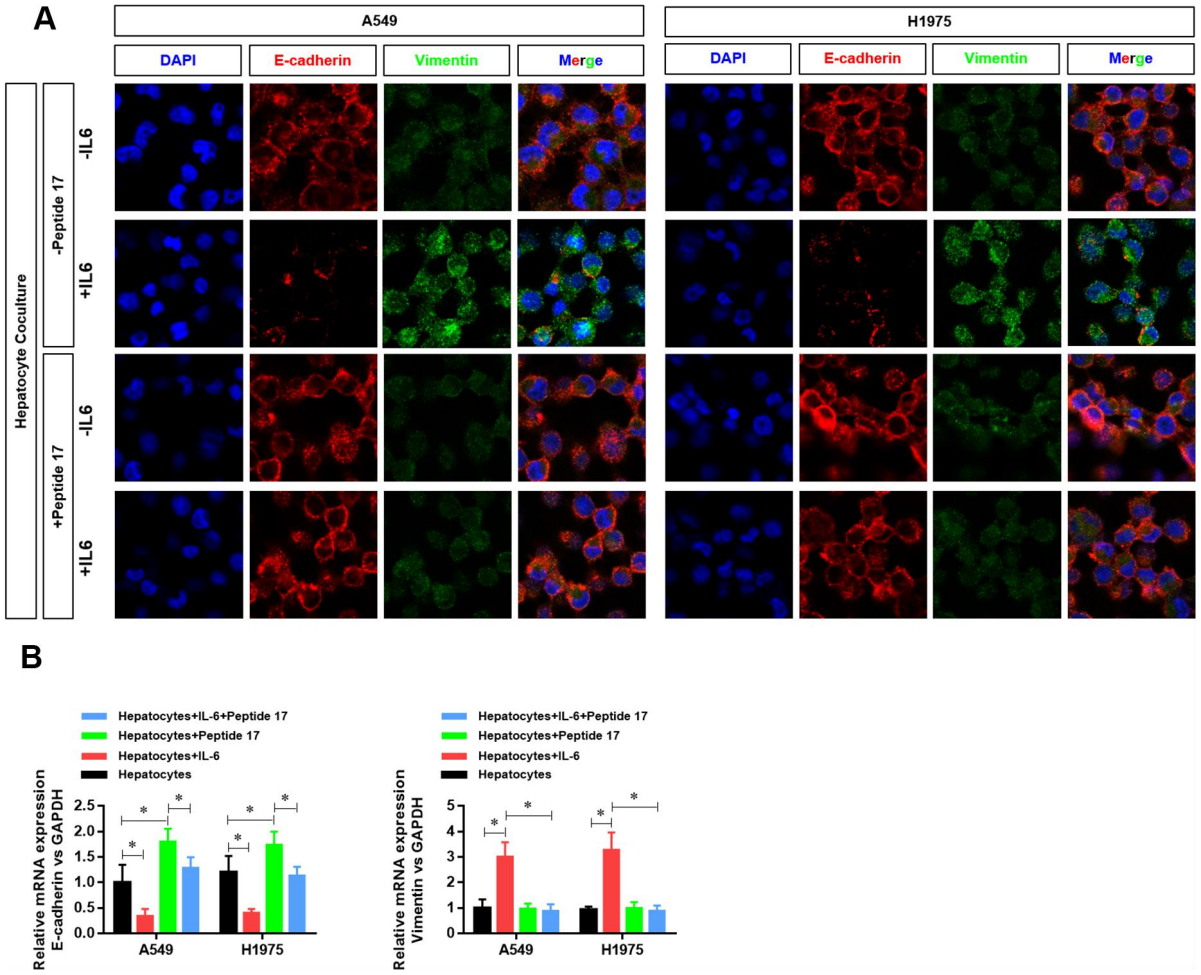
**YAP1 inhibition suppressed the ability of the hepatic inflammatory microenvironment to promote EMT.** (**A**) Changes in the expression of the EMT indexes E-cadherin and vimentin in A549 and H1975 cells were detected by confocal laser scanning microscopy (×630). (**B**) Changes in *CDH1* and *VIM* mRNA expression in A549 and H1975 cells were detected by real-time PCR (*P<0.05 versus the control group).

Treatment with peptide 17 had no significant effect on the protein (Figure 6A) and mRNA expression (Figure 6B) of E-cadherin and vimentin (indexes of EMT activation in LUAD cells) in the hepatic inflammatory microenvironment, demonstrating that the YAP1/TEAD signaling pathway plays an important role in the EMT activation of the inflammatory microenvironment in the liver.

### YAP1 inhibition significantly abolished the effects of the hepatic inflammatory microenvironment on the metastasis of LUAD cells *in vivo*

**Figure 7:**
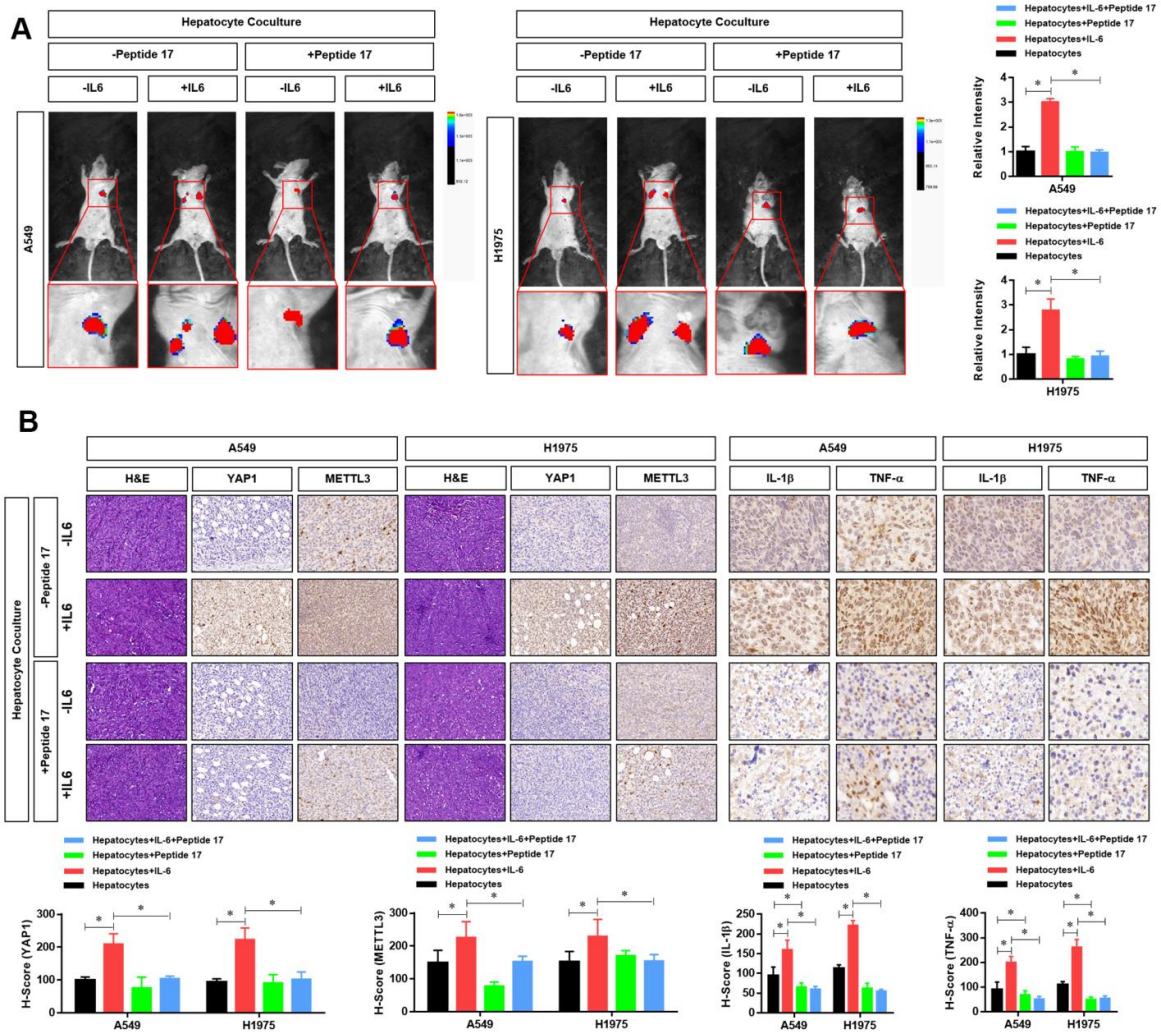
**YAP1 blockade significantly inhibited the effects of the hepatic inflammatory microenvironment on the metastasis of lung adenocarcinoma (LUAD) cells.** (**A**) Forty mice were divided into 8 groups, with 5 mice in each group. Cell grouping was similar to that of *in vitro* experiments, which were stimulated by the hepatic immune microenvironment and/or treated with the YAP1 inhibitor peptide 17 for 24 hours. After these treatments, 5×10^6^ cells each mouse was injected by tail vein and bioluminescence imaging (BLI) was performed 25 days later using the NightOWL II LB 983 imaging system (Berthold). (**B**) At the end of the experiment, all animals were sacrificed under anesthesia, and the metastasis tumors were fixated by Formalin and used for HE or IHC staining of YAP1 (×200), METTL3 (×200), IL-1b (×300) and TNF-a (×300). The quantitative analysis of IHC were performed by H-score scoring (*P<0.05 versus the control group).

Finally, we further investigated the role of the YAP1/TEAD signaling pathway *in vivo*. First, we constructed a metastasis model of nude mice injected with LUAD cells via the tail vein. The results showed that metastasis and tumorigenesis in the hepatic inflammatory microenvironment were significantly enhanced compared with that in the control group (Figure 7A). However, treatment with peptide 17 abolished the ability of the liver inflammatory microenvironment to promote the metastasis and tumorigenicity of LUAD cells, and there was no significant difference between the two groups (Figure 7A). Using immunohistochemistry, we further detected differences in the expression of METTL3 and YAP1 in the metastasis of LUAD cells. Similar to the results obtained *in vitro*, we found that the expression of METTL3 and YAP1 in LUAD cells was significantly increased in the hepatic inflammatory microenvironment. However, after treatment with peptide 17, METTL3 and YAP1 expression in the LUAD metastasis formed by the inflammatory liver microenvironment were not significantly increased, similar to the control group (Figure 7B). Moreover, we evaluated the protein level of two inflammatory cytokines (IL-1b and TNF-a) in the metastatic tumors. The results showed that the protein levels of IL-1b and TNF-a in the hepatocyte co-culture+IL-6 treated LUADs derived metastatic tumor were dramatically elevated, whereas significantly decreased in the hepatocyte co-culture+Peptide 17 treated LUADs. These results demonstrated that YAP1 inhibition significantly abolished the effects of the hepatic inflammatory microenvironment on the metastasis of LUAD cells *in vivo*.

## DISCUSSION

The tumor microenvironment affects the biological characteristics of tumors through several specific factors and signaling mechanisms [6]. In this study, we found that the inflammatory microenvironment of the liver significantly promoted the proliferation, migration, invasion, and EMT of LUAD cells [24]. In the tumor microenvironment, inflammatory cytokines, such as TNFα, TGFβ, IL-1β, IL-6, and IL-12, can regulate critical EMT transcription factors, including zinc finger E-Box-binding homeobox (ZEB1), ZEB2, the zinc finger protein Snail/Slug, and the helix spiral transcription factor Twist, thus initiating the EMT program [25]. The transcription factors ZEB and Snail suppress E-cadherin expression, and Twist decreases E-cadherin expression and induces N-cadherin expression, resulting in the loss of cell polarity, enhanced cell mobility, and tumor cell migration and invasion in epithelial cells [26]. In this study, we also found that the inflammatory microenvironment of the liver significantly inhibited the expression of E-cadherin and promoted the expression of vimentin in LUAD cells.

As the earliest identified RNA adenine methyl, m6A is the most abundant modified form of eukaryotic mRNA methylation, and it affects almost every stage of mRNA metabolism, including RNA folding, splicing, translation, and decay [18]. M6A methyltransferases (e.g., methyltransferase like 3-METTL3, METTL14, and METTL16), m6A demethyltransferases [e.g., fat mass and obesity-associated protein (FTO) and AlkB homolog 5 (ALKBH5)], and adaptive factors [e.g., Wilm’s tumor 1-related protein (WTAP), RNA binding motif protein 15 (RBM15), YTH domain (YTHs), and heterogeneous nuclear ribonucleoproteins (HNRNPs)] are involved in the progression of malignant tumors by recognizing m6A upon the reading, erasing, or writing of binding RNA in different tumors [20]. In this study, we found that the inflammatory microenvironment of the liver significantly increased m6A methylation and METTL3 expression in A549 and H1975 LUAD cells.

Hippo-Yes associated protein 1 (YES-associated protein 1, YAP1) was identified in 1994 by Sudol when the binding protein of the YES molecular regulatory region of SRC protein tyrosine kinase family members was isolated [27]. Studies show that YAP1 promotes cell proliferation, metastasis, survival, and stem cell activity [28, 29], which are critical processes in cancer cells. YAP1 is highly expressed in several solid tumors, such as HCC and gastric, colorectal, lung, breast, and pancreatic cancers, and is related to prognosis [30, 31]. In this study, we first found that METTL3 elevated the expression of *YAP1* in A549 and H1975 LUAD cells via m6A methylation. More importantly, we showed that the YAP1-specific inhibitor peptide 17 significantly inhibited the effects of the liver inflammatory microenvironment on the proliferation, migration, invasion, and EMT of LUAD cells and further confirmed that peptide 17 significantly inhibited the ability of the hepatic inflammatory microenvironment to promote the metastasis of LUAD cells *in vivo*.

To the best of our knowledge, this is the first study to show that the inflammatory microenvironment of the liver can promote the proliferation, metastasis, and EMT of LUAD cells via the METTL3-mediated m6A methylation of *YAP1*. In terms of m6A, this study elucidated the regulatory and molecular mechanism by which the hepatic inflammatory microenvironment regulates the biological function of LUAD cells, which provides a new research strategy for the early diagnosis, treatment, and prognosis of lung cancer.

## MATERIALS AND METHODS

### Antibodies and other reagents

Recombinant IL-6 protein (ab9627) and the anti-YAP1 antibody (ab52771) were purchased from Abcam (Cambridge, Massachusetts, USA). The horseradish peroxidase-labeled goat-anti-mouse/rabbit secondary antibody (K500711) was purchased from Dako (Glostrup, Denmark). Rabbit anti-human GAPDH (Sigma, St. Louis, Missouri, USA) was used for western blot analysis. An RNeasy Mini kit was purchased from Qiagen (Valencia, California, USA), and a SYBR Green Master Mix kit was obtained from TaKaRa (Dalian, China). RPMI-1640 medium and fetal bovine serum (FBS) were purchased from Gibco (Cambrex, MD).

### Cell lines and cell culture

Human A549 and H1975 LUAD cell lines were obtained from the Shanghai Institute for Biological Sciences, Chinese Academy of Sciences (Shanghai, China). In the presence of penicillin G (100 U/ml), streptomycin (100 μg/mL), and 2 mM L glutamine, the cell lines were cultured in DMEM supplemented with 10% FBS. Cells were incubated in 5% CO_2_ at 37° C.

### Determination of cell survival rate

According to the manufacturer’s instructions, the cell survival rate was evaluated using a cell counting kit-8 (CCK-8, Beyotime, Shanghai, China). A total of 5×10^3^ A549 and H1975 cells were inoculated into 96-well plates and incubated for 24, 48, and 72 h. CCK-8 reagent was added to each well 3 h before the end of incubation, and the absorbance at 450 nm was measured in each well using a microplate reader. Increases or decreases in the absorbance compared with the initial value indicated cell proliferation or death, respectively. Each experiment was repeated at least three times.

### Wound healing assay

The migration ability of A549 and H1975 cells was detected by a cell wound scratch assay. A549 and H1975 cells were cultured in six-well plates. When the cells reached 90% confluence, a 200-μL pipette tip was used to create a small wound area. The cells were washed twice with PBS and then incubated for 48 h in serum-free DMEM at 37° C with 5% CO_2_. Images were taken at two time points (0 and 24 h), and the wound width was measured using a BX50 microscope (Olympus) with a calibrated visual grid. The data from three independent experiments were averaged and expressed as a percentage of the original width.

### CRP, TNF-α, and IL-22 levels in cell supernatants

According to the manufacturer’s instructions, the levels of CRP, TNF-α, and IL-22 in the supernatant of A549 and H1975 cells were detected using an ELISA Kit (purchased from Abcam).

### Transwell analysis

As previously mentioned, transwell assays was used to assess the invasiveness of human lung cancer cells. Transwell assays were performed with Matrigel-coated transwell chambers (Corning, New York, USA). Cells from different groups (2×10^5^ cells/well) were serum-starved for 24 h and then placed in the upper chamber. Medium containing 10% FBS was placed in the lower chamber as a chemoattractant. After 48 h of incubation, cells that had migrated to the lower chamber were collected and resuspended, and the non-migrated cells were removed from the top chamber with cotton swabs. The migrated cells were fixed, stained with 0.1% crystal violet, and then photographed under a microscope (Olympus, Tokyo, Japan). Finally, the migrated cells were counted from three randomly selected fields.

### Real-time polymerase chain reaction (RT-PCR)

RT-PCR was used to detect the expression of mRNA in human LUAD cells. TRIzol reagent (Invitrogen, USA) was used to extract total RNA from various cell lines, and the ABI 7600 system (Applied Biosystems, USA) was used for PCR according to the manufacturer’s instructions. Human *GAPDH* was selected as the housekeeping gene to normalize the expression level of each target gene. The primer sequences were as follows:

**Table:** 

**Gene name**	**Upstream primer (5'-3')**	**Downstream primer (5'-3')**
Human *METTL3*	TAATGGCCGTTCTGTGCTCAT	TTCCCAGGTCAATGCTGACA
Human *YAP1*	CCCTCGTTTTGCCATGAACC	GTTGCTGCTGGTTGGAGTTG
Human *GAPDH*	TTCTTTTGCGTCGCCAGCC	TCCCGTTCTCAGCCTTGACG

The relative expression of each target gene was calculated by the 2^-ΔΔCT^ method.

### Western blot analysis

YAP1 protein expression in different cell models was determined by western blot analysis. Briefly, whole-cell extracts were prepared from 1×10^6^ cells using RIPA lysis buffer (50 mM Tris/HCl pH 7.4, 150 mM NaCl, 1% NP-40, 0.25% Na-acetylcholine, 1 mM EDTA, and a protease inhibitor cocktail). The cells were lysed on ice for 30 min, and the lysates were collected in microtubules and centrifuged at 4° C for 15 min at 12,000 rpm. Then, the supernatant was collected, and the protein concentration was determined using a BCA protein assay kit (Beyotime, Jiangsu, China). Equal amounts of denatured proteins were separated by SDS-PAGE and transferred to a PVDF membrane (Millipore, USA). The membrane was blocked with 5% skimmed milk powder in TBS-T (20 mM Tris, pH 7.4, 137 mM NaCl, and 0.05% Tween-20) at room temperature for 3 h and then incubated with the primary antibody at 4° C overnight. Subsequently, the blot was washed and incubated with the secondary antibody at room temperature for 1 h, followed by three washes with PBST. Finally, the Odyssey scanning system (Li-Cor, Lincoln, United States) was used to detect immunoreactive protein bands.

### Immunofluorescence

Cells were seeded in six-well plates at a density of 5×10^5^/ml, then cells were fixed with 4% paraformaldehyde for 15 min, washed with PBS, and permeabilized with 0.1% Triton X-100 for 10 min. After washing with PBS, cells were incubated with 1% BSA in PBS for 30 min. The cells were then incubated with anti-E-cadherin and anti-vimentin antibodies (both diluted in 1% BSA blocking buffer for 1 h). The cells were then incubated for 1.5 h with a suitable secondary antibody conjugated to Alexa Fluor 555 (Invitrogen). These cells were stained with phalloidin conjugated to Alexa Fluor 488 and DAPI to detect vimentin and cell nuclei, respectively. The coverslips were then sealed with 50% glycerin in PBS. Fluorescent images were captured with a Zeiss LSM510 confocal microscope equipped with a 63× oil immersion objective lens, NA 1.25 objective lens, and pinhole with a 0.1 Airy Disk. ImageJ software was used for image analysis.

### Total m6A measurements

An EpiQuik M6A RNA methylation quantitative detection kit was purchased from Epigentek Group (Farmingdale, New York) to quantitatively detect the level of m6A in total RNA according to the manufacturer’s specifications.

### Luciferase activity dependent on TEAD

To examine the luciferase activity dependent on TEAD, HK-2 cells were transfected with 200 ng of the pGL3-TEAD reporter and 10 ng pRL-TK (Promega) using Lipofectamine 3000 (Thermo Fisher). Then, luciferase activity was analyzed by the Dual-Luciferase Reporter Assay System (Promega).

### Methylated m6A RNA immunoprecipitation (Me-RIP) analysis

An anti-m6A antibody (ab151230; Abcam) was used for me-RIP. Methylated RNA was analyzed by qRT-PCR to detect methylated *YAP1* levels.

### Xenograft model analysis

Animal experiments were performed in strict accordance with the recommendations of the Guide for the Care and Use of Experimental Animals. The animal-related protocol was approved by the Animal Experimental Ethics Committee of the Affiliated Third Hospital of Suzhou University. All surgeries were performed under chloral hydrate anesthesia, and every effort was made to minimize the suffering of animals. Forty mice (six-week-old male BALB/c nude mice) were purchased from the Shanghai Laboratory Animal Research Center (Shanghai, China) and maintained in specific pathogen-free conditions. Forty mice were divided into 8 groups, with 5 mice in each group. Cell grouping was similar to that of *in vitro* experiments, which were stimulated by the hepatic immune microenvironment and/or treated with the YAP1 inhibitor peptide 17 for 24 hours. After these treatments, 5×10^6^ cells each mouse were injected by tail vein and bioluminescence imaging (BLI) was performed 25 days later using the NightOWL II LB 983 imaging system (Berthold). At the end of the experiment, all animals were sacrificed under anesthesia, and the metastasis tumors were fixated by Formalin and used for HE or IHC staining.

### Statistical analysis

Statistical analysis was performed using the paired Student t-test, Wilcoxon signed-rank test, chi-squared test, or log-rank survival analysis for the final analysis of data. The data are expressed as the mean and range or mean±SD of three independent experiments. All statistical analyses were performed using the GraphPad Prism 5.0 package (GraphPad software company, San Diego, USA). *P*-values of <0.05 were considered statistically significant.

### Data availability statement

There is no shared data.
